# Different associated factors of subjective cognitive complaints in patients with early and advanced Parkinson's disease

**DOI:** 10.3389/fnagi.2023.1257799

**Published:** 2023-12-01

**Authors:** Juan Huang, Lin Chen, Binbin Hu, Hui Wang, Xinyue Zhang, Xingfu Tian, Shu Wang, Wei Huang

**Affiliations:** ^1^Department of Neurology, Second Affiliated Hospital of Nanchang University, Nanchang, China; ^2^Department of Intensive Medicine, Second Affiliated Hospital of Nanchang University, Nanchang, China

**Keywords:** early Parkinson's disease, advanced Parkinson's disease, subjective cognitive complaints, nonmotor symptoms, attention

## Abstract

Subjective cognitive complaints (SCCs), defined as cognitive decline reported by subjects or their informants, are common in the early stage of Parkinson's disease (PD). Previous studies have shown a significant association between SCCs and non-motor features as well as objective cognitive decline in PD patients. However, the discrepancy in SCC prevalence and SCC-related factors between patients with early PD and those with advanced PD remains poorly understood. We recruited a total of 114 and 69 early PD patients and advanced PD patients, respectively. Univariate and multivariate logistic regression analyses were performed for early PD and advanced PD patients. The prevalence of SCCs in the early PD and advanced PD groups was 60.5 and 68.1%, respectively. In the early PD group, the presence of SCCs in early PD participants was significantly associated with a higher nonmotor symptoms questionnaire (NMSQ) score (OR = 1.05, 95% CI = 1.00–1.10, *p* = 0.040). SCCs in the advanced PD group were related to lower attention scores (OR = 0.24, 95% CI = 0.05–0.90, *p* = 0.043) and lower visuospatial/executive abilities scores (OR = 0.18, 95% CI = 0.04–0.86, *p* = 0.032). The prevalence and SCC-related factors are distinct in early PD and advanced PD. These findings suggest that SCCs in PD patients with different disease statuses appear to have different related factors that may depend on different disease severities.

## 1 Introduction

Parkinson's disease (PD) is a common neurodegenerative and movement disorder characterized by typical motor symptoms, including resting tremor, rigidity, bradykinesia, and postural instability. Approximately 40% of patients are estimated to present with mild cognitive impairment (MCI) at baseline, and ~75% of MCI patients progress to dementia in 10 years (Hely et al., 2005; Vu et al., 2012; Stuart et al., 2016; Obeso et al., 2017).

Subjective cognitive complaints (SCCs) in PD refer to cognitive impairment reported by the subjects themselves or their informants who may or may not have objective cognitive impairment (Barbosa et al., 2019). Studies have reported that the prevalence of PD patients with SCCs ranges from 31 to 60% (Lehrner et al., 2014; Baschi et al., 2018). Additionally, in a PD-SCC-related cross-sectional study, Dujardin et al. (2010) reported more objective cognitive impairment in PD patients with SCCs than those without SCCs. Koster et al. (2015) found that PD patients showed a higher proportion of self-reported dysfunction in attention and executive domains, and Baschi et al. (2018) showed a lower MoCA score in PD patients with SCCs than in those without SCCs. These two studies support each other and show a relationship between SCCs and decreased cognitive manifestation. A longitudinal study by Galtier suggested that PD patients with SCCs are at higher risk of converting to dementia than PD patients without SCCs (36.4 vs. 14.3%) 7.5 years later, and PD patients with SCCs showed dysfunction in visuospatial and visuoperceptual domains, which was associated with thinning of posterior cortices (Galtier et al., 2021, 2022). A follow-up study (1–7.5 years) led by Han et al. (2021) found that PD patients with SCCs showed memory impairment and greater reductions in attention and executive function than those without SCCs, which suggested that PD-SCCs may be high-risk individuals' later progression to cognitive decline. This result was consistent with Mills et al. (2020) and Purri et al.'s (2020) study, whose conclusion showed that PD with SCCs is more likely to develop into PD-MCI, and PD with SCCs may have the potential to predict cognitive decline later. In summary, it is important to explore SCC-related factors (including demographic features, motor performance, and non-motor symptoms), which may be beneficial to identify and manage PD with objective cognitive impairment as early as possible.

Most of the reported studies have suggested an association between mood features and SCCs (Purri et al., 2020; Chua et al., 2021; Han et al., 2021; Rosenblum et al., 2022; Yang et al., 2022). Chua et al. (2021) found an association between PD-SCCs and emotional factors (depression, anxiety, and apathy). According to Baschi et al.'s (2018) study, emotional disorders may be both a cause and a consequence of PD with SCCs. Siciliano et al. (2020) found that the prevalence of SCCs was higher in fatigued PD patients than in non-fatigued PD patients (35 vs. 9%). Another study reported an association between minor hallucinations and SCCs (Bejr-kasem et al., 2021). Since the SCCs in PD participants have the potential to further develop dementia, it is essential to explore unknown factors associated with SCCs.

Previous studies have shown increased severity and incidence of motor symptoms as well as non-motor symptoms with disease progression in PD patients (Sagna et al., 2014). However, the discrepancy in SCC prevalence and related factors between early and advanced PD patients remains unknown. Therefore, in this study, we aimed to explore the prevalence of SCC among patients with early PD and advanced PD and to further investigate SCC-related factors, such as demographic features, motor performance, and non-motor symptoms, which included age, sex, education, MDS-UPDRS-III, global cognitive function, fatigue, sleep, rapid eye movement sleep behavior disorder (RBD), depression, and anxiety, in the two groups separately.

## 2 Methods

### 2.1 Participants

The recruited participants were outpatients and inpatients at the Neurology Department of the Second Affiliated Hospital of Nanchang University from October 2021 through December 2022.

The inclusion criteria for the PD patients were as follows (Postuma et al., 2015): (a) those who met the PD diagnostic criteria of both the United Kingdom Brain Bank and Movement Disorders Society (MDS), (b) those who were stable on antiparkinsonian medication, and (c) those who were able to walk independently for at least 5 min. The exclusion criteria were as follows: (a) a history of psychiatric disorders or neurological diseases other than PD, (b) other neurological or musculoskeletal disorders that impair gait or coordination, and (c) severe dementia, anxiety, depression, or other medical conditions that resulted in an inability to complete the study.

### 2.2 Data collection

Demographic data (age, sex, and education) and disease duration were recorded. The Movement Disorders Society-Unified Parkinson's Disease Rating Scale (MDS-UPDRS) was collected, and the MDS-UPDRS part III and Hoehn–Yahr (H-Y) stages were used to evaluate motor function. According to Hoehn–Yahr stage, individuals with Hoehn–Yahr stage 1–2 were defined as early PD patients, and patients with Hoehn–Yahr stage 3–4 were classified as the advanced PD group. UPDRS Part I was used to define SCCs (Han et al., 2021; Xiao et al., 2021), and patients with a UPDRS-I 1.1 score *(1.1) cognitive* impairment ≥1 were regarded as PD patients with SCCs. Additionally, global cognitive function was assessed by the Montreal Cognitive Assessment (MoCA); visuospatial, naming, attention, language, abstraction, memory, and orientation domains were recorded to assess cognitive function in a more detailed manner. Anxiety and depression were measured by the Hamilton Anxiety Scale (HAMA) and Hamilton Depression Scale (HAMD), respectively. The Epworth Sleepiness Scale (ESS) was used to assess daytime sleep behavior disorders. The Pittsburgh Sleep Quality Index (PSQI) was used to measure sleeping quality. The Parkinson's Disease Questionnaire (PDQ-39) was adopted to evaluate quality of life. RBD was assessed by rapid eye movement sleep behavior disorder—Hong Kong (RBD-HK). PD-related fatigue symptoms as assessed by the Fatigue Severity Scale (FSS). Non-motor indicators were assessed by the Non-Motor Symptoms Scale (NMSS). PD-related automatic symptoms were assessed by the Scales for Outcomes in Parkinson's disease—Autonomic (SCOPA-AUT).

This study was approved by the Research Ethics Committee of the Second Affiliated Hospital of Nanchang University [Approval Review [2021] No. (69)]. Written informed consent was obtained from all subjects.

### 2.3 Data analysis

SPSS 25.0 (IBM Corporation, USA) was used to perform statistical analysis of the data. Demographic and clinical data were reported as frequency (percent) and mean (standard deviation)/median (1/4 quartile, 3/4 quartile), respectively, for categorical and continuous variables.

The Kolmogorov–Smirnov test was used to test the distribution of the collected data. Almost all variables except MoCA domains of naming, attention, language, abstraction, memory, and orientation domains showed normal distribution. Univariate and multivariate logistic regression analyses were first performed for all the samples. In univariate logistic regression, factors such as age, sex, disease duration, education, MDS-UPDRS I/II/III/total scores, total MoCA score, MoCA domain scores, FSS, NMSS, ESS, PQSI, RBD-HK, SCOPA-AUT, PDQ-39, HAMA, and HAMD were included as independent variables, and the presence of SCCs was regarded as a binary and dependent variable. Multivariate binary logistic regression analysis was used to investigate which variables were related to the presence of SCCs. Factors with a *P* ≤ 0.05 in the univariate regression analysis were subsequently used as independent variables, demographic characteristics (age, gender, and education) were regarded as control variables in multivariate logistic regression analysis, and the dependent variable was the presence of SCCs in the full sample. Then, we classified all the samples into early PD and advanced PD groups according to the Hoehn–Yahr stage. We determined factors with a *P* ≤ 0.05 in univariate binary regression analysis in the advanced PD group, and these factors were then included as independent variables in multivariate logistic regression analysis for the advanced PD group. The same protocol was performed in the early PD group. The regression model was processed with the backward stepwise regression method. Odds ratio (OR) values and 95% confidence intervals (95% CIs) were reported, and model validation was measured by the Hosmer–Lemeshow test. A *p* ≤ 0.05 was regarded as statistically significant.

## 3 Results

### 3.1 Demographic and clinical characteristics

We recruited a total of 114 and 69 early PD patients and advanced PD patients, respectively. Table 1 shows the demographic and clinical characteristics. A total of 69 (60.5%) early PD and 47 (68.1%) advanced PD patients reported SCCs. In the early PD group, the patients with SCCs manifested higher proportion of women (*p* = 0.026), lower education level (*p* = 0.011), higher scores in I/II/III part of UPDRS (*p* = 0.000, *p* = 0.001, *p* = 0.030), NMSS (*p* = 0.001), ESS (*p* = 0.008), SCOPA-AUT (*p* = 0.040), HAMA (*p* = 0.008), and HAMD (*p* = 0.001) and lower scores in the MoCA (*p* = 0.000), MoCA domains of visuospatial-executive abilities (*p* = 0.002), attention (*p* = 0.007), language (*p* = 0.012), memory (*p* = 0.001) than those without SCCs (Table 3). Additionally, advanced PD patients with SCCs showed significantly higher scores in UPDRS-I/II (*p* = 0.003, *p* = 0.048), NMSS (*p* = 0.006), SCOPA-AUT (*p* = 0.004), and HAMA (*p* = 0.045) and a lower score in the MoCA domains of visuospatial-executive abilities (*p* = 0.037), attention (*p* = 0.041), and memory (*p* = 0.046) than those without SCCs (Table 3).

**Table 1:** Demographic and clinical characteristics of early PD and advanced PD patients.

	**All (*n* = 183)**	**Early PD (*n* = 114)**	**Early PD with SCC (*n* = 69, 60.5%)**	**Early PD without SCC (*n* = 45, 39.5%)**	**Advanced PD (*n* = 69)**	**Advanced PD with SCC (*n* = 47, 68.1%)**	**Advanced PD without SCC (*n* = 22, 31.9%)**
Age^#^	64.63 ± 8.66	62.99 ± 8.48	64.08 ± 7.50	61.32 ± 9.66	67.34 ± 8.31	68.58 ± 8.06	64.68 ± 8.40
Sex(male/female)	105/78	69/45	36/33	33/12	36/33	24/23	12/10
Education(years)^#^	7.99 ± 4.35	8.70 ± 4.31	7.83 ± 4.06	10.00 ± 4.39	6.78 ± 4.17	6.31 ± 3.92	7.85 ± 4.61
Disease duration(years)^#^	5.17 ± 3.26	3.98 ± 2.91	3.75 ± 2.80	4.33 ± 3.07	7.30 ± 2.74	7.36 ± 2.49	7.14 ± 3.39
UPDRS I^#^	8.25 ± 6.01	6.26 ± 4.70	7.90 ± 5.05	3.76 ± 2.61	11.52 ± 6.52	13.23 ± 6.08	7.86 ± 6.02
UPDRS II^#^	11.81 ± 7.66	8.09 ± 5.49	9.46 ± 5.54	5.98 ± 4.73	15.87 ± 8.43	17.28 ± 7.51	12.86 ± 9.61
UPDRS III^#^	28.91 ± 19.25	22.46 ± 12.03	24.48 ± 12.76	19.38 ± 10.1	39.97 ± 21.96	41.74 ± 17.13	36.18 ± 29.93
UPDRS total^#^	48.44 ± 27.17	36.86 ± 18.43	41.91 ± 18.85	29.11 ± 14.89	67.57 ± 28.57	72.26 ± 23.08	57.55 ± 36.34
MoCA^**#**^	21.67 ± 5.60	22.99 ± 4.73	21.40 ± 4.61	26.11 ± 3.17	19.45 ± 6.28	18.15 ± 6.22	22.13 ± 5.69
Visuospatial/executive abilities^#^	3.45 ± 1.56	3.66 ± 1.48	3.27 ± 1.51	4.43 ± 1.10	3.08 ± 1.64	2.73 ± 1.65	3.81 ± 1.42
Naming^#^	3.00(3.00, 3.00)	3.00(3.00, 3.00)	3.00(3.00, 3.00)	3.00(3.00, 3.00)	3.00(2.00, 3.00)	3.00(2.00, 3.00)	3.00(2.00, 3.00)
Attention^#^	6.00(5.00, 6.00)	6.00(5.00, 6.00)	5.00(4.00, 6.00)	6.00(6.00, 6.00)	5.00(4.00, 6.00)	4.00(3.50, 5.50)	6.00(5.00, 6.00)
Language^#^	3.00(2.00, 3.00)	3.00(2.00, 3.00)	3.00(1.00, 3.00)	3.00(3.00, 3.00)	2.00(1.00, 2.00)	2.00(1.00, 3.00)	2.00(1.00, 3.00)
Abstraction^&^	1.00(0.00, 2.00)	1.00(0.00, 2.00)	1.00(0.00, 2.00)	2.00(1.00, 2.00)	1.00(0.00, 2.00)	0.00(0.00, 1.50)	1.00(0.25, 2.00)
Memory^&^	2.00(1.00, 3.00)	2.00(1.00, 3.00)	2.00(0.00, 3.00)	3.00(2.00, 4.00)	1.00(0.00, 2.00)	0.00(0.00, 2.00)	2.00(1.00, 2.75)
Orientation^#^	6.00(5.00, 6.00)	6.00(5.0, 6.0)	6.00(6.00, 6.00)	6.00(6.00, 6.00)	6.00(5.00, 6.00)	6.00(5.00, 6.00)	6.00(4.25, 6.00)
FSS^#^	42.20 ± 18.41	39.69 ± 16.91	42.06 ± 16.76	35.90 ± 16.65	46.35 ± 20.11	46.96 ± 20.63	44.95 ± 19.29
NMSS^#^	33.66 ± 23.72	24.10 ± 14.96	28.10 ± 15.30	17.71 ± 12.03	49.45 ± 26.98	55.76 ± 26.96	34.95 ± 21.23
ESS^#^	7.53 ± 5.32	6.59 ± 4.63	7.54 ± 4.97	5.07 ± 3.60	9.09 ± 6.01	9.22 ± 6.07	8.80 ± 6.01
PQSI^#^	8.05 ± 4.12	7.68 ± 4.01	8.13 ± 4.30	6.95 ± 3.42	8.65 ± 4.27	8.93 ± 4.28	8.00 ± 4.27
RBD-HK^#^	20.85 ± 18.24	18.48 ± 17.13	19.26 ± 16.09	16.90 ± 19.26	24.70 ± 19.46	26.03 ± 20.86	21.38 ± 15.51
SCOPA-AUT^#^	28.91 ± 7.70	26.31 ± 6.24	27.31 ± 6.84	24.63 ± 4.67	33.32 ± 7.98	35.36 ± 7.17	28.22 ± 7.79
PDQ-39^#^	29.06 ± 22.87	20.27 ± 15.33	23.97 ± 17.24	14.49 ± 9.30	46.49 ± 25.37	50.14 ± 28.35	38.06 ± 13.90
HAMA^#^	10.12 ± 7.24	7.49 ± 5.20	8.55 ± 5.41	5.82 ± 4.41	14.55 ± 8.04	15.87 ± 8.45	11.45 ± 6.11
HAMD^#^	8.29 ± 6.64	6.31 ± 5.50	7.74 ± 5.98	4.07 ± 3.60	11.63 ± 7.12	12.70 ± 7.72	9.10 ± 4.73

# mean ± SD;

& median(1/4quartile, 3/4 quartile).

UPDRS, *Unified* Parkinson's Disease Rating Scale; MoCA, Montreal Cognitive Assessment; FSS, Fatigue Severity Scale; NMSS, Non-Motor Symptoms Scale; ESS, Epworth Sleepiness Scale; PQSI, Pittsburgh sleep quality index; RBD-HK, Rapid eye movement sleep behavior disorder-HK; SCOPA-AUT, Scales for Outcomes in Parkinson's disease – Autonomic; PDQ-39, The Parkinson's Disease Questionnaire; HAMA, Hamilton Anxiety Scale; HAMD, Hamilton Depression Scale.

### 3.2 Factors associated with SCCs in all PD patients

First, a univariate logistic analysis of the whole sample was conducted. The presence of SCCs in all PD patients was used as a binary variable. Demographic features such as age, sex, education, and disease duration and non-motor symptoms such as MoCA scores and scores in its domains (visuospatial, naming, attention, language, abstraction, memory, and orientation), FSS, PQSI, HAMA, and HAMD were included in the univariate logistic analysis of all PD patients (*n* = 183). According to univariate logistic regressions, factors with *p* ≤ 0.05, such as age, sex, education, UPDRS-I/II/III, MoCA, and MoCA domains of executive abilities/attention/memory, NMSS, ESS, SCOPA-AUT, HAMA, and HAMD were associated with SCCs in all included PD patients (Supplementary Table 1). Subsequently, we conducted a multivariate logistic regression with factors with a *p* ≤ 0.05 in univariate logistic analysis. The results showed that SCCs were associated with lower visuospatial-executive abilities scores (OR = 0.24, *P* = 0.023), attention scores (OR = 0.26, *p* = 0.032), and memory scores (OR = 0.31, *P* = 0.019) when adjusted by age, sex, and education in all PD samples (Table 2).

**Table 2:** Associated factors of SCCs in all PD patients.

	**AdjOR**	**95%CI**	***p*-value**
Visuospatial/executive abilities	0.24	0.07–0.82	**0.023**
Attention	0.26	0.08–0.88	**0.031**
Memory	0.31	0.12–0.82	**0.019**

Visuospatial/executive abilities, attention, memory: domain of Montreal Cognitive Assessment (MoCA); AdjORs, ORs have been adjusted by sex, age, education; Statistically significant differences (p < 0.05) are shown in bold.

### 3.3 Factors associated with SCCs in early PD and advanced PD patients

We analyzed patients in the early PD and advanced PD subgroups separately. First, a univariate logistic analysis including all demographic features and non-motor symptoms was performed in early PD and advanced PD. In the advanced PD patients, factors such as UPDRS-I/II, NMSS, SCOPA-AUT, HAMA, and MoCA domains of visuospatial-executive abilities, attention, and memory showed a probable association with SCCs (*p* < 0.05; Table 3), and these factors were then included in the multivariate binary logistic regression analysis of the advanced PD group. The results showed that SCCs in the advanced PD group were related to lower attention scores (OR = 0.24, 95% CI = 0.05–0.90, *p* = 0.043) and lower visuospatial/executive abilities scores (OR = 0.18, 95% CI = 0.04–0.86, *p* = 0.032; Table 4). Similarly, we analyzed early PD with the same protocol. The results suggested that a higher NMSS score (OR = 1.05, 95% CI = 1.00–1.10, *p* = 0.040) was associated with SCCs in early PD patients (Table 4).

**Table 3:** Factors associated with SCCs in the early PD and advanced PD patients in univariate logistic regressions.

	**Early PD**			**Advanced PD**		
	**OR**	**95%CI**	***p*** **-value**	**OR**	**95%CI**	***p*** **-value**
**Demographic characteristics**						
Age	1.04	0.99–1.09	0.093	1.06	0.99–1.13	0.073
Sex	0.40	0.18–0.89	**0.026** ^*^	0.87	0.32–2.40	0.870
Education	0.88	0.80–0.97	**0.011** ^*^	0.91	0.80–1.04	0.173
Disease duration	0.93	0.81–1.08	0.350	1.03	0.82–1.30	0.798
UPDRS I	1.40	1.20–1.64	**0.000** ^*^	1.18	1.06–1.32	**0.003** ^*^
UPDRS II	1.14	1.05–1.24	**0.001** ^*^	1.08	1.00–1.15	**0.048** ^*^
UPDRS III	1.04	1.00–1.08	**0.030** ^*^	1.01	0.99–1.04	0.326
UPDRS total	1.05	1.02–1.08	**0.001** ^*^	1.02	0.99–1.04	0.050^*^
**Non motor symptoms**						
MoCA	0.70	0.58–0.84	**0.000** ^*^	0.89	0.79–0.99	0.044^*^
Visuospatial/executive abilities	0.46	0.28–0.76	**0.002** ^*^	0.62	0.39–0.97	0.037^*^
Naming	0.54	0.18–1.66	0.283	0.58	0.29–2.01	0.575
Attention	0.35	0.16–0.75	**0.007** ^*^	0.58	0.33–0.97	0.041^*^
Language	0.32	0.13–0.78	**0.012** ^*^	0.76	0.43–1.32	0.328
Abstraction	0.43	0.23–0.83	**0.011** ^*^	0.51	0.25–1.05	0.066
Memory	0.54	0.37–0.78	**0.001** ^*^	0.64	0.42–0.99	0.046^*^
Orientation	0.18	0.03–1.24	0.081	0.92	0.58–1.45	0.700
FSS	1.02	0.99–1.05	0.067	1.01	0.98–1.03	0.708
NMSS	1.06	1.03–1.09	**0.001** ^*^	1.04	1.01–1.06	0.006^*^
ESS	1.14	1.03–1.25	**0.008** ^*^	1.01	0.93–1.11	0.794
PQSI	1.08	0.98–1.20	0.136	1.06	0.93–1.20	0.412
RBD-HK	1.10	0.98–1.04	0.535	1.01	0.98–1.05	0.418
SCOPA-AUT	1.08	1.00–1.16	**0.040** ^*^	1.16	1.05–1.29	0.004^*^
PDQ-39	1.06	1.02–1.10	**0.004** ^*^	1.02	0.99–1.05	0.120
HAMA	1.12	1.03–1.22	**0.008** ^*^	1.09	1.00–1.18	0.045^*^
HAMD	1.19	1.07–1.32	**0.001** ^*^	1.09	0.99–1.20	0.064

* p < 0.05; UPDRS, unified Parkinson's disease rating scale; MoCA, Montreal cognitive assessment, FSS, fatigue severity scale; NMSS, non-motor symptoms scale; ESS, epworth sleepiness scale; PQSI, Pittsburgh sleep quality index; RBD-HK, rapid eye movement sleep behavior disorder-HK; SCOPA-AUT, scales for outcomes in Parkinson's disease—autonomic; PDQ-39, the Parkinson's disease questionnaire-39; HAMA, Hamilton anxiety scale; HAMD, Hamilton depression scale.

**Table 4:** Associated factors of SCCs in early PD patients and advanced PD patients.

	**AdjORs**	**95%CI**	***p*-value**
**Early PD**			
NMSS	1.05	1.00-1.10	**0.040**
**Advanced PD**			
Visuospatial/executive abilities	0.18	0.04-0.86	**0.032**
Attention	0.24	0.05-0.90	**0.043**

NMSS, non-motor symptoms scale; Visuospatial/executive abilities, attention, domain of Montreal Cognitive Assessment (MoCA); AdjORs, ORs have been adjusted by sex, age, education; Statistically significant differences (p < 0.05) are shown in bold.

## 4 Discussion

In our study, we investigated the prevalence of SCCs and their related factors, including demographic features, motor symptoms, and non-motor symptoms, among all PD patients as well as PD patients with different disease stages. The results of this study indicated that 63.4% of all PD patients reported SCCs. In addition, patients with SCCs were older, had a higher proportion of women, and had lower education levels. The presence of SCCs in all PD patients had a significant association with the performance of visuospatial-executive abilities, attention, and memory. Subsequently, we focused on PD patients with different disease stages (early PD and advanced PD); 60.5% of early PD patients and 68.1% of advanced PD patients reported SCCs. Additionally, the patients with SCCs in early PD showed those who had more NMSS were susceptible to experiencing SCCs, while advanced PD patients who had worse attention and visuospatial-executive abilities were more likely to report SCCs. Our results showed that SCCs in PD patients have different associations that are dependent on underlying disease severity.

In this study, we showed that 63.4% of PD patients subjectively complained of SCCs, which is higher than the value found in Erro et al.'s (2014) and Xiao et al.'s (2021) studies. The reasons for this discrepancy may be as follows: First, older age as well as longer disease duration contribute to a higher prevalence of SCCs as studies have suggested that the prevalence of SCCs increases with age (Taylor et al., 2018). Additionally, the score of the cognitive complaints interview (CCI), which is a scale used to measure SCCs, increased with the duration of PD (Hong et al., 2018). A study by Erro reported a 30.3% prevalence of subjective memory complaints (SMCs) in PD patients; the mean age was younger (58.2 ± 8.2 years old), and the disease duration was shorter (<2 years) than in our study. Moreover, assessment tools for SCCs should also be responsible for different SCC incidences. Memory complaints alone were considered and recorded in Erro's research; however, the “non-amnestic” pattern is considered more common in the PD group according to another study (Dupouy et al., 2018), which might lead to a lower presence of SCCs if the study focuses only on memory complaints. Finally, the definition of SCCs influences the results. Xiao showed that 22.3% of 332 PD patients had SCCs; however, patients here were at normal objective cognition assessed by neuropsychological tests. We defined SCCs as memory/thinking impairment or/with disorientation and executive dysfunction or worse cognitive problems according to UPDRS-I 1.1. There were some other different results between previous studies and our study. For example, a study led by Barbosa et al. (2019) demonstrated that 85% of the PD cohort reported experiencing SCCs, which was a significantly higher proportion of SCCs than ours. It is worth noting that the PD patients were older, had a longer disease duration, and had lower MoCA scores than our patients. A study described that poorer cognitive performance was more frequent among PD patients with SCCs (Dujardin et al., 2010). Therefore, individuals with older age, longer duration, and poorer cognitive status will be more prone to have complaints about cognitive function.

When we divided all samples into early PD and advanced PD groups, 60.5% of early PD patients and 68.1% of advanced PD patients reported SCCs. The prevalence of SCCs in early PD patients was higher than that in Hong et al. (2014) and Purri et al.'s (2020) results (53 and 54.3%, respectively) and a subjective measurement tool including more cognitive domains may be responsible for the discrepancy here. UPDRS-I 1.1, used by our study, includes memory, attention, thinking, and orientation domains, while in Hong and Purri's results, a single yes/no question (“Do you feel that you have a declining memory?”) was used to define SCCs. The proportion of SCCs in early PD was similar to the 60% of SCCs in Baschi et al.'s (2018) research. In Baschi's study, demographic characteristics such as age and education were similar to ours, which had similar cohort features to ours. Therefore, discrepancies in the prevalence of SCCs in PD patients among studies might be associated with cohort demographic characteristics (i.e., age and disease duration), the definition of SCCs, and assessment tools for SCCs.

In early PD patients, the cohort with SCCs exhibited a significant association with the performance of the non-motor symptoms (NMS), which is consistent with other study results (Erro et al., 2012; Pan et al., 2021). Erro et al. showed that the presence of NMSS domain 5 was common in PD patients; logistic regression results in Pan's study revealed that PD with SCCs was significantly related to the Non-Motor Symptoms Scale score (OR = 1.340). The NMSS assesses nine non-motor domains, ranging from sleep dysfunction, fatigue, subjective cognitive decline, and mood to sexual dysfunction, and a higher NMSS score is associated with quality of life, which may remind individuals of their cognitive dysfunction (Pan et al., 2021). Hence, our results showed that a higher NMSS score is related to SCCs in early PD patients and may reflect cognitive impairment. NMSS-domain 5 evaluates memory/attention by three questions (memory, loss of interest, and concentrating), and SCCs were often identified as NMSS-domain≥1 (Barbosa et al., 2019). Therefore, a high consistency between UPDRS-I 1.1 and NMSS-domain 5 to identify SCCs may partly contribute to SCC-related higher NMSS scores in early PD in our study. More detailed NMSS scores in nine domains separately may help to accurately identify the association between NMSS and SCCs.

In advanced patients, the related factors for SCCs were different from those in the early PD group. We found that attention and visuospatial-executive abilities were SCCs-associated factors in advanced PD patients, which is consistent with the results of some studies (Hong et al., 2012; Koster et al., 2015; Mills et al., 2016; Baschi et al., 2018; Dupouy et al., 2018; Han et al., 2021; Yang et al., 2022). Hong et al. (2012) showed that PD patients with SCCs manifested poorer performance in attention-associated tasks; Han et al. (2021) suggested that SCCs may be an indicator of attention dysfunction in PD. Mills et al. (2016) showed that changes in visuospatial-executive performance had a significant impact on PD patients with SCCs. According to neuroimaging results, executive functions are dominated by the frontal lobe, the parietal lobe, and the anterior cingulate gyrus, which are responsible for attention, and attention in PD patients is associated with dopaminergic hypofunction in the caudate nucleus (Jokinen et al., 2009; Hong et al., 2012). Mills et al. (2016) reported that early cognitive complaints were associated with posterior cortical deficits in PD patients. Therefore, poor performance of attention and visuospatial-executive abilities in advanced PD with SCCs may be robust, and SCCs in advanced PD patients may reflect potential PD-related pathology to some extent.

In our study, we found a probable association between emotional factors and PD patients (including all PD patients, early and advanced PD patients' group) in the univariate regression analysis, while the relationship was lost when adjusted factors by sex, age, and education. The results were in line with other studies that demonstrated no relationships between SCCs and emotional features (Hong et al., 2012; Dupouy et al., 2018). However, there were discrepancies between our results and those of other studies such as some other studies reporting affective factors were associated with PD-SCCs (Koster et al., 2015; Baschi et al., 2018; Yang et al., 2022). Koster et al. (2015) suggested that it is essential to take anxiety into consideration when evaluating cognitive complaints. Baschi et al. (2018) reported that PD with SMC was significantly associated with anxiety (OR = 3.93), and Yang et al. (2022) showed that PD-SCD was significantly associated with HAMA scores. Since the relationship between mood features and PD-SCCs is unclear now, the definition of SCCs and sample heterogeneity (inclusion/exclusion criteria, age, disease duration, and cognitive status) may be responsible for the discrepancy, and more studies are required to explore the association.

## 5 Limitations

There are several limitations in our study. First, a UPDRS-I 1.1 score of ≥1 evaluating SCCs was too simple compared with a comprehensive screening scale. However, SCCs identified by UPDRS-I 1.1 have been revealed to be associated with the development of PD-MCI, which validates the clinical significance of our measurement tool. Second, we used a short MoCA scale to assess global cognition rather than a comprehensive neuropsychological test focused on each cognitive domain. It is necessary to confirm our results with comprehensive neuropsychological tests. Furthermore, a larger sample and follow-up studies are essential to validate our cross-sectional results.

## 6 Conclusion

In conclusion, our study showed that the prevalence and SCC-related factors are distinct in early PD and advanced PD. There was a higher prevalence of SCCs in advanced PD than in early PD, suggesting that the disease stage might influence the presence of SCCs. Early PD patients who had higher NMSS scores were more likely to report experiencing SCCs. Better attention and visuospatial-executive abilities measured by MoCA were associated with better self-reported cognitive function in advanced PD patients. The results of our study suggested that different SCC-associated factors in early PD and advanced PD are dependent on underlying disease severity.

Consequently, the report on SCCs, which is simple to collect, has clinical significance and reminds clinicians to pay attention to some SCC-related factors and help to better manage PD patients.

## Data availability statement

The raw data supporting the conclusions of this article will be made available by the authors, without undue reservation.

## Ethics statement

The studies involving humans were approved by the Research Ethics Committee of the Second Affiliated Hospital of Nanchang University [Approval Review (2021) No. (69)]. The studies were conducted in accordance with the local legislation and institutional requirements. The participants provided their written informed consent to participate in this study.

## Author contributions

JH: Conceptualization, Writing – original draft. LC: Software, Writing – review & editing. BH: Data curation, Writing – review & editing. HW: Project administration, Resources, Writing – review & editing. XZ: Project administration, Resources, Writing – review & editing. XT: Project administration, Resources, Writing – review & editing. SW: Writing – review & editing. WH: Conceptualization, Funding acquisition, Writing – review & editing.
