# Supplementary material for: Different associated factors of subjective cognitive complaints in patients with early and advanced Parkinson's disease
Source: Front Aging Neurosci. 2023 Dec 1;15:1257799. doi: 10.3389/fnagi.2023.1257799 (PMC10722415; doi:10.3389/fnagi.2023.1257799)
Supplement: Supplementary file 1 [file Table_1.docx]

**Table S1** Factors associated with SCCs in PD patients in univariate logistic regressions

|  | **OR** | **95%CI** | **p-value** |
| --- | --- | --- | --- |
| **Demographic characteristics** |  |  |  |
| Age | 1.05 | 1.01-1.09 | **0.010*** |
| Sex | 0.52 | 0.28-0.98 | **0.043*** |
| Education | 0.89 | 0.83-0.96 | **0.002*** |
| Disease duration | 1.01 | 0.91-1.12 | 0.900 |
| UPDRS I | 1.23 | 1.13-1.33 | **0.000*** |
| UPDRS II | 1.10 | 1.04-1.15 | **0.000*** |
| UPDRS III | 1.02 | 1.00-1.04 | **0.000*** |
| UPDRS total | 1.03 | 1.01-1.04 | **0.000*** |
| **Non motor symptoms** |  |  |  |
| MoCA | 0.82 | 0.75-0.90 | **0.000*** |
| Visuospatial | 0.54 | 0.39-0.75 | **0.000*** |
| Naming | 0.66 | 0.32-1.33 | 0.242 |
| Attention | 0.49 | 0.32-0.75 | **0.000*** |
| Language | 0.58 | 0.38-0.87 | **0.009*** |
| Abstraction | 0.47 | 0.29-0.75 | **0.002*** |
| Memory | 0.59 | 0.45-0.78 | **0.000*** |
| Orientation | 0.73 | 0.45-1.17 | 0.188 |
| FSS | 1.02 | 0.99-1.03 | 0.074 |
| NMSS | 1.04 | 1.02-1.06 | **0.000*** |
| ESS | 1.08 | 1.01-1.15 | **0.022*** |
| PQSI | 1.07 | 0.99-1.16 | 0.075 |
| RBD-HK | 1.01 | 0.99-1.03 | 0.284 |
| SCOPA-AUT | 1.10 | 1.05-1.16 | **0.000*** |
| PDQ-39 | 1.03 | 1.01-1.05 | **0.002*** |
| HAMA | 1.10 | 1.04-1.16 | **0.001*** |
| HAMD | 1.13 | 1.06-1.20 | **0.000*** |

***p < 0.05；UPDRS**：Unified Parkinson’s Disease Rating Scale；**MoCA：**Montreal Cognitive Assessment；**FSS：**Fatigue Severity Scale；**NMSS：**Non-Motor Symptoms Scale；**ESS：**Epworth Sleepiness Scale；**PQSI：** Pittsburgh sleep quality index； **RBD-HK：**Rapid eye movement sleep behaviour disorder-HK；**SCOPA-AUT：**Scales for Outcomes in Parkinson's disease - Autonomic；**PDQ-39：**[The Parkinson's Disease Questionnaire](https://www.parkinsons.org.uk/professionals/resources/parkinsons-disease-questionnaire-pdq-39)；**HAMA：**Hamilton Anxiety Scale；**HAMD：**Hamilton Depression Scale
